# Self-organization of computation in neural systems by interaction between homeostatic and synaptic plasticity

**DOI:** 10.1186/1471-2202-16-S1-O5

**Published:** 2015-12-18

**Authors:** Sakyasingha Dasgupta, Christian Tetzlaff, Tomas Kulvicius, Florentin Wörgötter

**Affiliations:** 1 Institute for Physics - Biophysics, Georg-August-University, D-37077 Göttingen, Germany; 2 Bernstein Center for Computational Neuroscience, Georg-August-University, D-37077 Göttingen, Germany; 3 Maersk-Moller Mckinsey Institute, Southern Denmark University, Odense, Denmark

## 

**Figure 1:**
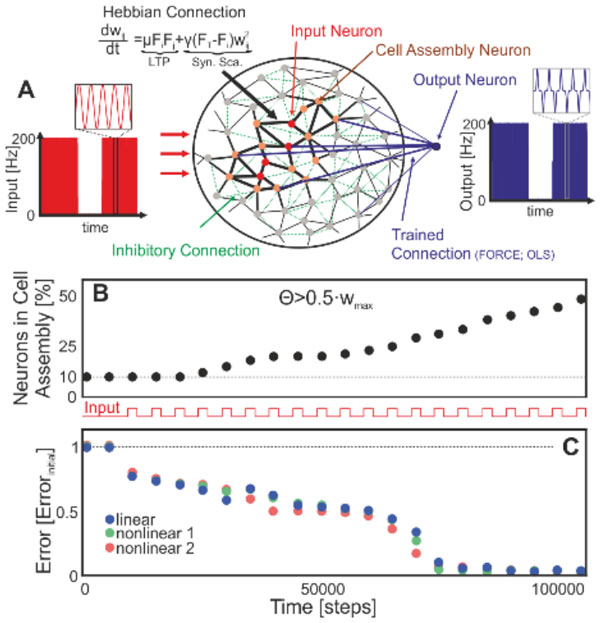
**Cell assembly size and computational performance are correlated**. (A) Input-driven formation of cell assemblies brought about by the interaction long-term potentiation (LTP) and synaptic scaling (Syn. Sca.). (B) With more learning trials the assembly grows and integrates more neurons. We measure this by arbitrarily defining assembly size by that set of neurons connected with efficacies larger than half the maximum weights. (C) Parallel to the outgrowth of the cell assembly the error of the system to perform several linear and non-linear calculations decreases.

The ability to perform complex motor control tasks is essentially enabled by the nervous system via the self-organization of large groups of neurons into coherent dynamic activity patterns. During learning, this is brought about by synaptic plasticity, resulting in the formation of multiple functional networks - commonly termed 'cell-assemblies'. A multitude of such cell assemblies provide the requisite machinery for non-linear computations needed for the mastery of a large number of motor skills. However, given the fact that there exists considerable overlap between the usage of the same neurons within such assemblies, for a wide range of motor tasks, creation and sustenance of such computationally powerful networks poses a challenging problem. How such interwoven assembly networks self-organize and how powerful assemblies can coexist therein, without catastrophically interfering with each other remains largely unknown. One the one side, it is already known that networks can be trained to perform complex nonlinear calculations [1], such that, if the network possesses a reservoir of rich, transient dynamics, desired outputs can be extracted from these reservoirs in order to enable motor control. On the other side, cell assemblies are created by Hebbian learning rules that strengthen a synapse if pre- and post-synaptic neurons are co-active within a small enough time window [2]. Therefore it appears relatively straightforward to combine these mechanisms in order to construct powerful assembly networks. However, given that the self-organization of neurons into cell assemblies by the processes of synaptic plasticity induces ordered or synchronized neuronal dynamics, which can destroy the required complexity of a reservoir network, such a combination remains a very challenging problem [3]. Furthermore, simultaneous creation of multiple cell assemblies can also lead to catastrophic interference if one cannot prevent them from growing into each other. In this study, we exploit for the first time the interaction between neuronal and synaptic processes acting on different time scales to enable, on a long time scale, the self-organized formation of assembly networks (Fig. 1), while on a shorter timescale, to conjointly perform several non-linear calculations needed for motor fine-control. Specifically, by the combination of synaptic plasticity and synaptic scaling [4], as a homeostatic mechanism, we demonstrate that such self-organization allows executing a difficult, six degrees of freedom, manipulation task with a robot where assemblies need to learn computing complex nonlinear transforms and - for execution - must cooperate with each other without interference. This mechanism, thus, permits for the first time, the guided self-organization of computationally powerful sub-structures in dynamic networks for behavior control. Furthermore, comparing our assembly network to networks with unchanging synapses ("static" networks) shows that it is indeed the embedding of a strongly connected assembly that creates the necessary computational power.
